# Towards interoperability in infection control: a standard data model for microbiology

**DOI:** 10.1038/s41597-023-02560-x

**Published:** 2023-09-23

**Authors:** Eugenia Rinaldi, Cora Drenkhahn, Benjamin Gebel, Kutaiba Saleh, Hauke Tönnies, Friederike D. von Loewenich, Norbert Thoma, Claas Baier, Martin Boeker, Ludwig Christian Hinske, Luis Alberto Peña Diaz, Michael Behnke, Josef Ingenerf, Sylvia Thun

**Affiliations:** 1 Berlin Institute of Health, Charité Universitätsmedizin, Berlin, Germany; 2 Institute of Medical Informatics (IMI), University of Lübeck, Lübeck, Germany; 3 Klinik für Infektiologie und Mikrobiologie, Universitätsklinikum Schleswig-Holstein, Campus Lübeck, Lübeck, Germany; 4 Data Integration Center, Jena University Hospital, Jena, Germany; 5 Universitätsklinikum Münster, Münster, Germany; 6 Institute of Virology, University of Mainz, Mainz, Germany; 7 Institute for Hygiene and Environmental Medicine, Charité Universitätsmedizin, Berlin, Germany; 8 Hannover Medical School, Institute for Medical Microbiology and Hospital Epidemiology, Hannover, Germany; 9 Technische Universität München, München, Germany; 10 Institute for Digital Medicine, University Hospital Augsburg, Augsburg, Germany

**Keywords:** Medical research, Health care

## Abstract

The COVID-19 pandemic has made it clear: sharing and exchanging data among research institutions is crucial in order to efficiently respond to global health threats. This can be facilitated by defining health data models based on interoperability standards. In Germany, a national effort is in progress to create common data models using international healthcare IT standards. In this context, collaborative work on a data set module for microbiology is of particular importance as the WHO has declared antimicrobial resistance one of the top global public health threats that humanity is facing. In this article, we describe how we developed a common model for microbiology data in an interdisciplinary collaborative effort and how we make use of the standard HL7 FHIR and terminologies such as SNOMED CT or LOINC to ensure syntactic and semantic interoperability. The use of international healthcare standards qualifies our data model to be adopted beyond the environment where it was first developed and used at an international level.

## Introduction

### The need for coordination in infection control

The increased number of global infectious diseases outbreaks including the recent COVID-19 pandemic^1^, together with the increasing burden of antimicrobial resistance^2^, call for a more coordinated approach across different countries in this field. Currently, different institutions often use heterogeneous data structures and idiosyncratic local codes to report similar results. This requires a time-consuming process of data harmonization before being able to merge and jointly analyze the data^3^. To facilitate data exchange and integration between institutions, healthcare IT standards are needed to univocally define both structure and semantics of data elements. Mappings from proprietary codes to standard codes are inherently error-prone and generally result in lower data quality. Ideally, standard codes should already be used in primary clinical record systems, either directly or indirectly via continuously maintained mappings in the system (early mapping). Using standards follows the FAIR principles that aim at ensuring data remain findable, accessible, interoperable and reusable^4,5^. Furthermore, the use of interoperability standards enables local or national data models to potentially be used by a wider scientific community across institutions and countries.

Within the Medical Informatics Initiative (MII)^6^, all German university hospitals have agreed to define a common and standard-based interoperable set of information relevant to patient care, the so-called MII Core Data Set (CDS). The MII CDS has been organized into several modules, each specifying the core information of a specific patient–relevant topic such as person, procedure or diagnosis. Within each module, a dedicated group of experts, including university representatives knowledgeable in healthcare IT and/or relevant medical domains, decides on the specific information that is pertinent to the module and studies its relations and junctures both intra- and inter-modularly. Additionally, information needs to be modeled within the group according to the healthcare IT standards recommended by the MII National Steering Committee both for structure and semantics. A microbiology module was foreseen as part of the CDS to describe the procedures performed in laboratories on any possible sample collected from in or outpatients, to detect, identify and characterize microorganisms and their properties. This module can play a vital role in the scientific community as it, if commonly applied, facilitates a cross-institutional study of infectious diseases and antimicrobial resistance. Our multidisciplinary working group has shaped the microbiology module to standardize the related information. The goal is to offer one common format for the microbiology data that can be publicly adopted across different institutions and to provide a common international terminology in place of, or together with, the local codes to enhance interoperability.

### Interoperability standards and existing data models

In 2017, the MII National Steering Committee produced a first document, later updated, containing a high-level description of the CDS as well as recommendations on the syntactic and semantic standards to be employed in all modules^7^. On the structural level, information should be modeled using the Fast Healthcare Interoperability Resources (FHIR)^8^, a recent Health Level Seven (HL7)^9^ standard rapidly gaining adoption for the exchange of healthcare information^10–12^. It is organized in several resources (e.g. Patient, Observation, Diagnosis) which can be customized to obtain context-specific profiles. On the semantic level, international terminologies such as SNOMED Clinical Terms (SNOMED CT)^13^ and Logical Observation Identifiers Names and Codes (LOINC)^14^ are recommended; for numerical results, Unified Code for Units of Measure (UCUM)^15^ standard units should be used. SNOMED CT is the most comprehensive general-purpose terminology in the field of healthcare with about 350,000 unique concepts organized in hierarchies; LOINC is a terminology system specific for laboratory and measurement information. UCUM is a code system which includes all units of measures internationally used in science, engineering, and business. To deal with the apparent overlap between the terminologies, we adhered as much as possible to existing best practices as stated by LOINC and SNOMED International^16^.

Since the general idea behind the MII is to improve interoperability of data, the re-use of available standard data models from relevant international or national projects such as AKTIN^17^ or the International Patient Summary^18^ should also be considered. In the case of microbiology, we used the thematically related laboratory CDS module as starting point as well as the information model already developed for the use case Infection Control (IC) of the HiGHmed^19,20^ consortium within the MII. Additional impulses incorporated into the modeling process came from the HELP^21^ use case of another MII consortium, SMITH^22^, dedicated to the use of antibiotics in infection medicine and from the guidelines of the Austrian electronic health record (ELGA)^23^. In general, we built on previous experiences but expanded the scope to comprise all possible microbiology examinations including microbial genetics. We also implemented international standards for both the structure and the semantics of the data with the aim to create a model that could be applied at global level.

## Results

### The UML diagram

One of the main results of the working group was the definition of a core set of microbiology data elements to be shared among university hospitals, which includes the most relevant information pertaining to the common investigations in the field.

**Fig. 1:**
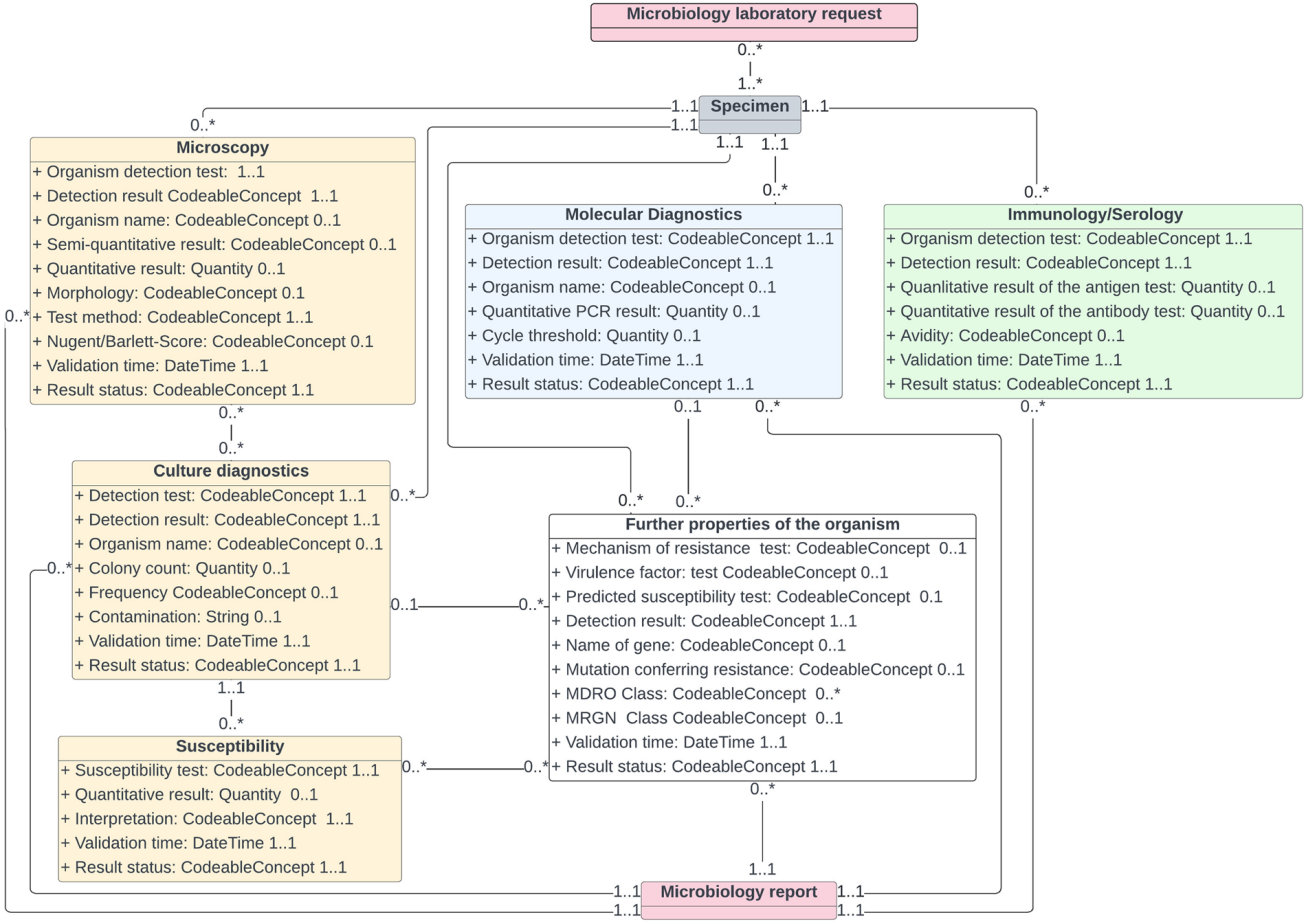
UML Diagram for microbiology information.

Figure 1 (also available in higher definition in Supplementary File 1) shows the organization of the information in a Unified Modeling Language (UML)^24^ diagram and the relationships between the different segments. The microbiology module itself references other modules of the MII CDS such as those related to patient or to encounter. For simplicity, we decided to include only the reference to the module related to specimen in the UML as it appeared particularly relevant.

As the UML diagram shows, three main blocks of information were defined: the yellow boxes represent the culture-related information; the blue and the green ones represent molecular diagnostics and immunology/serology respectively. A fourth group, represented in the white box, includes tests that explore other properties of the organism using multiple techniques. The pink boxes represent information concerning the request to the laboratory and its subsequent report. The gray box is a reference to the MII-Module Biobank, which contains the profile for specimen. FHIR profiles and value sets (VS) for the elements of the four groups of information have been developed and made publicly available; their descriptions are provided here below.

### FHIR profiles and terminology bindings

The following paragraphs and tables offer an overview of the FHIR profiles and terminology bindings that have been established as a result of our work. They are by no means an exhaustive representation of the entire information model, but rather provide a summary of the main elements and of their binding to a fixed value (FV) or VS of the referenced terminology. Among VSs we can distinguish between extensional and intensional ones, abbreviated in the following tables with VSe and VSi; the first ones being an enumerated list of specific codes, the second ones being a group of codes algorithmically defined according to a specific rule^25,26^. For VSs that contain an extensive list of elements, we provided the direct link to the resource on a public repository.

#### Culture diagnostics

##### Microorganism detection, colony count and susceptibility

This block of information describes examinations to detect microorganisms through culture and to observe their characteristics. Following the GECCO^27^ data model, a SNOMED CT VS containing the concepts “detected, not detected, inconclusive” was chosen to constrain the primary result in the FHIR *Observation.value* element. The FHIR element describing the test, *Observation.code*, was bound to the LOINC code: *11475-1 Microorganism identified in Specimen by Culture* as shown in Table 1.

**Table 1:** Microorganism detection via culture. FHIR Observation elements profiled to describe the detection of a microorganism via culture.

FHIR element	FV/VS	Code(s)	Description	Terminology
Observation.code	FV	11475-1	Microorganism identified in Specimen by Culture	LOINC
Observation.value	VSe	260373001	Detected (qualifier value)	SNOMED CT
		260415000	Not detected (qualifier value)	SNOMED CT
		419984006	Inconclusive (qualifier value)	SNOMED CT
Observation.component.value	VSi	<410607006 (with exceptions)	Organism (organism) hierarchy with exceptions	SNOMED CT

To offer a standard terminology for the names of the microorganisms, the SNOMED CT hierarchy of *410607006 |Organism (organism)|* was selected. However, considering that microbiological cultures are focused only on the organisms causing diseases, the VS only includes elements from the subhierarchies of Domain Bacteria, Virus, Prion, Kingdom Fungi, and Kingdom Protozoa. Conversely, the sub-groups Domain Archaea, Kingdom Animalia, Kingdom Plantae, Kingdom Viridiplantae, Slime mold, were not considered to be relevant for the microbiology module^28,29^.

As illustrated in Table 2, a VS of four alternative LOINC codes representing colony counts was specified to meet the need for different units in the laboratories. Additionally, some laboratories prefer to report a semi-quantitative result (e.g., *Present+ out of++++*) rather than a numerical value. In FHIR, both possibilities can be offered as alternatives so that the choice is left to the laboratory.

**Table 2:** Colony count. FHIR Observation elements profiled to describe the colony count.

FHIR element	FV/VS	Code(s)	Description	Terminology
Observation.code	VSe	49223-1	Colony count [#/volume] in Unspecified specimen by Visual count	LOINC
		564-5	Colony count [#] in Unspecified specimen by Visual count	LOINC
		38436-2	Colony count [#/mass] in Unspecified specimen by Visual count	LOINC
		20774-6	Colony count [Units/volume] in Unspecified specimen by Visual count	LOINC
Observation.value			Numerical result with units	UCUM
Observation.value	VSe	https://simplifier.net/medizininformatikinitiative-modullabor/laborergebnis-semiquantitativ	Semi-quantitative result	SNOMED CT

All specific antimicrobial susceptibility tests are described using the Observation resource with an antimicrobial-specific LOINC term as *Observation.code*, e.g., *18945-6 Methicillin [Susceptibility]*. In this specific case, the code does not contain any information concerning the methodology used, which can be entered separately in the *Observation.method*. However, some LOINC susceptibility test codes already include information on the methodology such as agar (disc) diffusion, MIC (minimum inhibitory concentration), gradient strip, MLC (minimum lethal concentration) and method for slow-growing mycobacteria. In this case information on the method does not need to be entered separately. Table 3 shows the intensional VS created to include these susceptibility tests expressed using the LOINC syntax.

**Table 3:** Susceptibility tests. FHIR Observation elements profiled to describe the susceptibility tests.

FHIR element	FV/VS	Code(s)	Description	Terminology
Observation.code	VSi	Property:Susc and method NOT Genotyping	Susceptibility tests not using the genotyping method	LOINC
Observation.value		Numerical result with units		UCUM
Observation.interpretation	VSe	S	Susceptible	EUCAST
		I	Resistant	EUCAST
		R	Intermediate/ susceptible -increased exposure	EUCAST
	VSe	S	Susceptible	HL7
		SDD	Susceptible Dose Dependent	HL7
		I	Intermediate	HL7
		R	Resistant	HL7
		NS	Nonsusceptible	HL7
Observation.interpretation.CodeableConcept.coding.version			String describing the version	—

The MIC and the MLC determination give a numerical result modeled in *Observation.value* with UCUM units *µg/mL, mg/L, [arb’U]/mL*. The *interpretation* element is useful to record the qualitative rating of the respective susceptibility which can be reported according to EUCAST^30^ or CLSI^31^ standards. As both committees have changed their definitions over the years, it is mandatory to enter the standards’ version used to report the susceptibility category^32,33^.

The values for interpretation S, I, R refer to the EUCAST categories: S stands for susceptible, R for resistant, I for intermediate or for susceptible -increased exposure according to the version of EUCAST being considered^33^. In the case of CLSI the VS consists of 5 values^34^: S for Susceptible, SDD for susceptible dose dependent, I for intermediate, R for resistant, NS for nonsusceptible.

##### Microscopy

The microscopic examination on the other hand, although not necessarily part of a culturing, was grouped in this block as it is often used to describe culture results. It is represented by a SNOMED CT procedure code as shown in Table 4. The *Observation.value* element describes, by means of the SNOMED CT terminology, whether a microorganism was detected or not.

**Table 4:** Microscopy. FHIR Observation elements profiled to describe the microscopy examination.

FHIR element	FV/VS	Code(s)	Description	Terminology
Observation.code	FV	117259009	Microscopy (procedure)|	SNOMED CT
Observation.value	VSe	260373001	Detected (qualifier value)	SNOMED CT
		260415000	Not detected (qualifier value)	SNOMED CT
		419984006	Inconclusive (qualifier value)	SNOMED CT
Observation.method	VSe	58586006	Microbial ova-parasite examination (procedure)|	SNOMED CT
		117023006	Thick film peripheral blood smear (procedure)	SNOMED CT
		408195004	Thick film for malarial parasites (procedure)	SNOMED CT
		117024000	Thin film peripheral blood smear method (procedure)	SNOMED CT
		67047002	Microbial wet smear (procedure)	SNOMED CT
		27318003	Potassium hydroxide preparation (procedure)	SNOMED CT
		104157003	|Light microscopy (procedure)	SNOMED CT
		73512001	Electron microscopic study (procedure)	SNOMED CT
	VSi	<127790008	Staining method (procedure) hierarchy	SNOMED CT
Observation.component.value	VSi	<410607006 with exceptions	Organism (organism) hierarchy with exceptions	SNOMED CT
Observation.component.value			Numerical result with units	UCUM
Observation.component.value	VSe	https://simplifier.net/medizininformatikinitiative-modullabor/laborergebnis-semiquantitativ	Semi-quantitative result	SNOMED CT
Observation.component.code	FV	75371-5	Bartlett score of Sputum Qualitative by Light microscopy	LOINC
Observation.component.code	FV	43391-2	Bacterial vaginosis score	LOINC

In this context, the *Observation.method* element contains essential information by specifying which method was used to obtain the results. There are several observation techniques that are commonly used in microscopy and we created a VS containing the methods described in the LOINC Microbiology Guide^35^ (e.g., 104157003 |Light microscopy (procedure)|, 67122001 |Acid fast stain method (procedure)|). Considering the variety of different procedures specifying the staining method, we included the entire hierarchy below the SNOMED CT concept *127790008 |Staining method (procedure)|*.

Other relevant results of the microscopic examination are the quantitative and semi-quantitative results, and the morphology of the microorganisms (e.g., Gram-positive cocci in chains). Additionally, the Bartlett score which is applied to grade the quality of sputum specimens^36^, and the Nugent score, employed to diagnose bacterial vaginosis^37^ were also considered. All these elements are modeled as *Observation.component* because they result from the same microscopic procedure.

#### Molecular diagnostics

##### Microorganism detection

As the second building block, the molecular diagnostics profiles describe the examinations occurring in microbiology to detect or identify microorganisms based on their genetic material. This block is also suitable for results generated by next generation sequencing.

In this case, as described in Table 5, the LOINC code *92253-4 Microorganism identified in Isolate or Specimen by Molecular genetics method* describes the observation, whereas for the result a SNOMED CT VS with the following options is proposed: *positive, negative, weakly positive, inconclusive*. The *observation.value* is a qualitative result confirming the presence or absence of the targeted genetic material.

**Table 5:** Molecular Diagnostics. FHIR Observation elements profiled to describe the detection of an organism via molecular diagnostics.

FHIR element	FV/VS	Code(s)	Description	Terminology
Observation.code	FV	92253-4	Microorganism identified in Isolate or Specimen by Molecular genetics method	LOINC
Observation.value	VSe	10828004	Positive (qualifier value)	SNOMED CT
		260408008	Weakly positive (qualifier value)	SNOMED CT
		260385009	Negative (qualifier value)	SNOMED CT
		419984006	Inconclusive (qualifier value)	SNOMED CT
Observation.component.value	VSi	<410607006 with exceptions	Organism (organism) hierarchy with exceptions	SNOMED CT
Observation.component.code	FV	398545005	Nucleic acid assay (procedure)	SNOMED CT
Observation.component.code	FV	9718006	Polymerase chain reaction analysis (procedure)	SNOMED CT

Similarly to the profile for the detection via culture, the name of the specific microorganism is specified as *Observation.component*. Related numerical results such as the number of cycles at which the target was detected^38–40^ and the amount of virus present are represented as components as well.

#### Immunology/serology

The third block describes the examinations that are needed to detect and characterize the presence of antibodies or antigens in a specimen. One profile was created and customized as shown in Table 6 with a general code in *Observation.code* to describe immunologic tests. However, the same element can be further specified using a LOINC code (e.g. *88603-6 Adenovirus Ag [Presence] in Lower respiratory specimen by Immunoassay)* chosen from the corresponding, ad-hoc created, VS. The qualitative result is expected to be reported using the same VS as in molecular diagnostics. If an additional numerical result^41,42^ is present, it can be expressed through the *Observation.component* data element.

**Table 6:** Serology/immunology. FHIR Observation elements profiled to describe the immunologic and serologic examination.

FHIR element	FV/VS	Code(s)	Description	Terminology
Observation.code	FV	25231800	Immunology laboratory test (procedure)	SNOMED CT
	VSe	https://simplifier.net/medizininformatik-initiative-modul-mikrobiologie/mii-vs-mikrobio-serologie-immunologie-loinc	Microbiology tests detecting the presence of antibodies or antigens	LOINC
Observation.value	VSe	10828004	Positive (qualifier value)	SNOMED CT
		260408008	Weakly positive (qualifier value)	SNOMED CT
		260385009	Negative (qualifier value)	SNOMED CT
		419984006	Inconclusive (qualifier value)	SNOMED CT
Observation.component.value			Numerical result of antigenic test with units	UCUM
Observation.component.value			Numerical result of antibody test with units	UCUM
Observation.component.code	FV	77559007	Immunologic avidity, function (observable entity)	SNOMED CT
Observation.component.value	VSe	75540009	High (qualifier value)	SNOMED CT
		62482003	Low (qualifier value)	SNOMED CT
Observation.component.value			Numerical result with units	UCUM

Also information concerning avidity, the strength of binding between an antibody and its specific epitope belongs to this block. This examination has been identified with the SNOMED CT code *77559007 |Immunologic avidity, function (observable entity)|* and the following SNOMED CT answer options: *62482003 |Low (qualifier value)|*, *75540009 |High (qualifier value)|*.

Also in this case, possible numerical results are defined through the *Observation.component* element.

#### Further properties of the organism

Based on susceptibility testing results, information concerning multi-drug resistance of the organism (MDRO) can be deduced. In particular, the MDRO characterization can be expressed by specifying what substances the microorganism is resistant to^43–46^. To this purpose, we managed to have a new concept included in SNOMED CT: 1285113001 |Type of antimicrobial resistant organism (observable entity)| which could be used as *Observation.code* as shown in Table 7. The corresponding susceptibility category of microorganisms was found to be codeable in detail only in the case of bacteria and possible values were identified using the SNOMED CT subhierarchy of *409793007 |Antimicrobial resistant bacteria (organism)|*. A vast variety of child concepts are available such as *707497007 |Carbapenem resistant bacteria (organism)|* which in turn are further split up into species-specific resistance classes like, in this case, 726492000 |*Carbapenem resistant Pseudomonas aeruginosa (organism*)*|*.

**Table 7:** Multidrug-resistant organism type. FHIR Observation elements profiled to describe the resistance type.

FHIR element	FV/VS	Code(s)	Description	Terminology
Observation.code	FV	1285113001	Type of antimicrobial resistant organism (observable entity)	SNOMED CT
Observation.value	VSi	<409793007	Antimicrobial resistant bacteria (organism) hierarchy	SNOMED CT
		<409795000	Antimicrobial resistant virus (organism) hierarchy	SNOMED CT
		<409794001	Antimicrobial resistant fungi (organism) hierarchy	SNOMED CT

In contrast, the concepts *409795000 |Antimicrobial resistant virus (organism)|* and *409794001 |Antimicrobial resistant fungi (organism)|*, do not have any child concepts in SNOMED CT at the moment. However, as further classification of the resistance type might be added in the future, we left this possibility open in our model.

The multi-drug resistant Gram-negative bacteria (MRGN)^47^ classification published by the Commission for Hospital Hygiene and Infection Prevention (KRINKO) at the Robert Koch Institute^48^, is commonly used in Germany for the purpose of hospital hygiene^48^ and was also included in the module (Table 8). The MRGN categories refer to different recommended measures of infection control based on the clinical relevance of the resistance pattern.

**Table 8:** MRGN classification by the Robert Koch Institute. FHIR Observation elements profiled to describe the MRGN type.

FHIR element	FV/VS	Code(s)	Description	Terminology
Observation.code	FV	99780-9	Multidrug resistant Gram-negative organism classification [Type]	LOINC
Observation.value	VSi	2MRGN	LA33214-0	LOINC
		3MRGN	LA33215-7	LOINC
		4MRGN	LA33216-5	LOINC

The values 2MRGN, 3MRGN, and 4MRGN indicate that an organism is resistant towards 2, 3 or 4 of the 4 antibiotic groups: ureidopenicillins, 3rd/4th generation cephalosporins, carbapenems, and fluoroquinolones. The detection of carbapenemases automatically leads to a classification as 4MRGN irrespective of the antimicrobial susceptibility testing results. The same holds true if carbapenems are tested resistant (except for *P. aeruginosa*)^49^. The 2MRGN value is solely used in neonatology^50^.

The data model concerning the mechanism of resistance^51^ describes the observation to detect specific resistance genes or mutations and is shown in Table 9. The element *Observation.code* is associated to the LOINC VS created to include only examinations searching for such resistance genes or mutations, e.g., *72421-1 Vancomycin resistance vanB gene [Presence] by Molecular method*.

**Table 9:** Mechanism of resistance. FHIR Observation elements profiled to describe the presence of a specific mechanism of resistance.

FHIR element	FV/VS	Code(s)	Descriptiom	Terminology
Observation.code	VSe	https://simplifier.net/medizininformatik-initiative-modul-mikrobiologie/mii-vs-mikrobio-resistenzgene-loinc	Tests demonstrating the presence of resistance genes or mutations.	LOINC
		https://simplifier.net/medizininformatik-initiative-modul-mikrobiologie/mii-vs-mikrobio-resistenzmutation-loinc	Class: Antibiotic susceptibilities (ABXBACT) and Property: Presence or Threshold (Prthr), Presence or identifier (Prid)	
Observation.value	VSe	10828004	Positive (qualifier value)	SNOMED CT
		260408008	Weakly positive (qualifier value)	SNOMED CT
		260385009	Negative (qualifier value)	SNOMED CT
		419984006	Inconclusive (qualifier value)	SNOMED CT
Observation.component			Name of the gene or mutation	NCBI/EMBL-EBI

If the gene or mutation under consideration is not yet included in any LOINC code, general codes can be used such as:

*92251-8 Microorganism gene detected [Presence] by Molecular method*

*92246-8 Microorganism resistance mutation detected [Presence] by Molecular method*

In this case, the name of the gene or mutation can be separately specified in *Observation.component* using a microbial genomics database such as that of the National Center for Biotechnology Information (NCBI) or the European Molecular Biology Laboratory/ European Bioinformatics Institute (EMBL-EBI).

Besides antimicrobial resistance, a pathogen’s virulence factor is also of particular interest for determining its harmfulness as they are involved for example in pathogen-adhesion, pathogen-invasion or immune evasion^52,53^. The VS created for tests that identify virulence factors includes LOINC codes describing microbiology examinations looking for specific proteins and toxins. As in the previous paragraphs, detection results are reported in *Observation.value* (Table 10).

**Table 10:** Virulence factor. FHIR Observation elements profiled to describe the presence of a specific virulence factor.

FHIR element	FV/VS	Code(s)	Descriptiom	Terminology
Observation.code	VSe	https://simplifier.net/medizininformatik-initiative-modul-mikrobiologie/mii-vs-mikrobio-virulenz-loinc	Microbiology tests looking for proteins, toxins, exotoxins and endotoxins.	LOINC
			Class: Microbiology (MICRO) and Property: Presence or Threshold (Prthr), Presence or identifier (Prid)	
Observation.value	VSe	260373001	Detected (qualifier value)	SNOMED CT
		260415000	Not detected (qualifier value)	SNOMED CT
		419984006	Inconclusive (qualifier value)	SNOMED CT

For all the profiles described above, the type of observation is specified by the LOINC code *18725-2 Microbiology studies (set)* in the FHIR element *Observation.category*.

A DiagnosticReport profile was also created to report microbiology results using the MII Laboratory module as a base profile. The type of report was specified in the *Observation.category* element using 4341000179107|Microbiology report| and the VS for microbiology studies (92894-5 Microbiology - bacterial studies, 92893-7Microbiology - viral studies, 96397-5 Microbiology - mycobacteriology studies, 96398-3 Microbiology - mycology studies, 92892-9 Microbiology - parasitic studies) in SNOMED and LOINC, respectively.

## Discussion

The proposed data model builds on the experiences gained in existing projects such as the MII use case IC^54^ but expands its scope to cover all clinically relevant organisms, and to include common microscopic examinations and genomic analyses^55^. Additionally, it also aims at a wide international adoption. The emerging FHIR standard was used to mold necessary elements of information into an interoperable data structure, as recommended by the MII government and by other relevant international authorities. The European Centre for Disease Prevention and Control (ECDC) for example, mentions FHIR as the standard to achieve standardization and interoperability for the effective sharing of data^56^. This notion is shared by the American Centers for Disease Control and Prevention (CDC), which calls for more efficiency in data exchange by adopting FHIR^57^.

The described FHIR profiles are publicly available on the web via the FHIR collaboration platform Simplifier together with a narrative Implementation Guide in German and English. The MII National Steering Committee has opened a commentary phase in which a larger group of MII participants and international stakeholders, will be able to provide their comments or requests for change. Following this phase, and after adjustments, the module will be tested with mock data on a project-a-thon event. On this occasion, all MII university hospitals will be invited to test the model with their data sets thereby identifying if further changes are needed.

### Modelling challenges

Clinical microbiology is a complex domain requiring profound expertise to understand the intricacies of relevant procedures, results, and their interrelations. Thus, a close collaboration between microbiologists and digital health specialists was a prerequisite for establishing a correct data model but there remains some potential for error or inaccuracy. Likewise, the model offered is not the only possible, and additionally, some changes might still occur.

Specific choices operated by the modelers can certainly be further discussed within wider expert groups also at international level. For instance, the choice between highly specific or more general codes to describe examinations can be debatable. We opted to use a generic LOINC code for the detection of a microorganism via culture and to separately employ SNOMED CT to code the specific organism name as *Observation.component*. In fact, FHIR allows the use of the component element to include additional results from the same examination. It could be argued that more precise LOINC terms which already include the name of the organisms should be employed thus avoiding to specify the names of the microorganisms in a separate component section. However, to allow the description of all microorganisms including those not (yet) considered in LOINC, the current modeling seemed to us the most efficient way to ensure a unique pattern of information. Additionally, this approach states clearly whether a pathogen was detected.

Additionally, diverging from the previously mentioned best practices, it was not always possible to use a LOINC code to describe the examination in *Observation.code*. In such cases SNOMED CT was used and requests were submitted to LOINC to create new codes. Should new more suitable codes become available, we would welcome such new solution in future versions of our model.

To reflect the variety of methodologies used for microscopic examination, as mentioned in the LOINC Microbiology Guide, we decided to use the Procedure hierarchy of SNOMED CT concepts. This turned out to be the most comprehensive way to model the information. However, also this choice might have to be reviewed in the future because it is planned to code all LOINC laboratory methods in the qualifier value hierarchy.

The decision of not binding the EUCAST values to SNOMED CT might also be challenged, as the terms used, seemingly, exist as concepts in the terminology. However, since the specific definitions vary across systems (e.g., EUCAST, CLSI) and because SNOMED CT does not explicitly refer to any of them, we decided to create a new specific EUCAST code system that removes any possible ambiguities. We intend to submit the drafted code system to EUCAST for endorsement.

The field of genetics, probably due to its more recent and fast evolving implementation compared to culture-based diagnostics or microscopy, shows an apparent lack of standardization. In the last fifteen years, when DNA next generation sequencing started to become available, biologists began to face the challenges of Big Data^58^. This process led to the creation of many databases mainly focused on human genomics as more and more centers developed their own sequencing capabilities. Some of these projects^59–63^ address viral, bacterial and fungal genetics as well.

Examinations that aim at identifying mechanisms of resistance or virulence are highly dependent on molecular methods, but Standard Development Organizations sometimes struggle to keep up with the fast evolving world of genetics. For reporting gene names or genetic information, we decided to propose the databases provided by the National Center for Biotechnology Information at the National Institutes of Health in the United States and the European Molecular Biology Laboratory/ European Bioinformatics Institute since SNOMED CT lacks appropriate coverage. These databases seemed very comprehensive, and additionally offer information from several interconnected databases.

Since virulence factors include a wide variety of molecules such as toxins, enzymes, lipopolysaccharides, surface structures such as capsules, it is difficult to create a VS or to define rules in LOINC to group them. A possibility could be to use a generic definition for the test such as “detection of virulence factor” and separately specify the respective virulence factor. However, the only available generic LOINC code would restrict the methodology to mouse bioassay which would exclude other methodologies. Additionally, it might be challenging to provide a unique source to describe information about toxins as they tend to be dispersed and of limited accessibility. However, initiatives such as Toxin and Toxin-Target Database (T3DB)^64^ are helping to overcome this problem. Submitting a request for a new general LOINC term will be evaluated. For the time being, starting from the list of codes that the microbiology working group considers particularly important, we selected in LOINC all the microbiology tests referring to proteins and toxins. We remain open to expand this VS both based on future discussions and via collaborations at international level.

In general, it appears clear there is not only one possible model to represent the data in FHIR. Modelers make their choices based on both FHIR specifications and personal judgment. For example, the Danish Microbiology Model group described their approach to FHIR in 2017^65^ as ‘close to their original model as possible’ to facilitate data migration. However, this approach led to the creation of many ad hoc extensions of the profiles, which presumably make the resulting FHIR model very specific to the local needs. Further, the model does not offer bindings to standard terminology. In our case, the choices of the modelers were dictated mainly by the goal of building an interoperable model based on FHIR specifications and on standard terminology that could be used across different institutions. We managed to map all the required information to FHIR without using extensions. A possible drawback of our model is the scarcity of mandatory elements, which was determined by the ambition to make it applicable to most institutions.

Most laboratories within the MII still use their own local lists and the mapping required to conform to the standard terminologies included in the model will certainly be challenging. However, it will enable them to efficiently exchange data both within the MII and also with any institution using the same terminology. By promoting the use of international terminology codes that can be adopted by any laboratory around the world the model implicitly discourages the use of local codes that hinder interoperability. However, if required, local codes could still be used in addition to the international ones to ease the transition.

### General applicability of the model

Increasing numbers of infectious disease outbreaks and ever-growing antimicrobial resistance^66^ require global collaboration, and the worldwide exchange and comparison of data will benefit from a standardized way of collecting information. Our proposed data model is designed to include all possible microbiology methods that might be of interest for any hospitals. However, it does not dictate which examinations should be performed. For example, the German MRGN classification is not required internationally and therefore, also the relative FHIR profile would not be used by all hospitals. In our opinion, the main challenges for any organizations adopting the model would be the same that we encountered: the slow process of replacing local codes with standard codes in laboratory information systems, and the slow implementation of a new FHIR infrastructure. Additionally, a license for the usage of SNOMED CT should be acquired if the country where the model is being implemented is not a SNOMED member.

We already presented part of our work at the Infectious Disease Workshop hosted by the Global Alliance for Genomics and Health (GA4GH)^67^ and by the Public Health Alliance for Genomic Epidemiology (PHA4GE)^68^. Additionally, we were invited by X-eHealth, the European Project focused on interoperable, secure and cross border electronic health record exchange, to present our work and join efforts. We intend to further strengthen these collaborations to improve the data model by verifying its usability also in other countries while supporting the diffusion of a common model. An interesting field of international collaboration could entail the modeling of microbiology information not yet included in the present model such as molecular typing.

Since infectious diseases coupled with antimicrobial resistance represents one of the top challenges to global health, and belong to the WHO core priorities^69–72^, it is of paramount importance to seek agreement on a common data model at an international level thus facilitating data integration, analysis and fostering research capabilities worldwide.

## Methods

The data model was developed in a two-step process. At first, a widely standard-agnostic logical model was agreed upon, and in a second step, concrete FHIR profiles and VSs were developed. To reduce redundancy and to emphasize their interrelations, both steps are described conjointly in this paper.

A group of twenty experts in digital health or in microbiology was invited to collaborate in regular web meetings in a 2-year time span. The participants belonged to different German universities and contributed in their respective roles of subject matter experts, data modelers, terminology experts or FHIR implementers as well as HELP and HiGHmed representatives.

### The building of a data sets

In order to create the logical model for the CDS microbiology module, the existing MII-related projects (laboratory module, HiGHmed and HELP) were analyzed to check if their results could be useful for our purpose. Additionally, the implementation guide provided by ELGA was examined as it contains a very detailed section dedicated to laboratory findings.

To ensure MII intra-modular interoperability, we decided to use the profiles created within the laboratory module of the CDS as base profiles. This means that all the profiles developed within the microbiology module are developed as further customizations of the laboratory module profiles. Initially, to facilitate the discussion on the choice of the data elements to be included in the microbiology module, we analyzed the HiGHmed data set. We listed all the HiGHmed data elements in a shared document and reviewed them individually during the biweekly group meetings. This way we included all the most relevant and commonly used information related to microbiology laboratory investigations and excluded elements that were out of scope. The IC use case for example, also covers information related to the patient´s stay in a healthcare organization which was not in the focus of the CDS microbiology module. For every element we collaboratively examined:

- Relevance to the microbiology module
- Completeness of information concerning the most used methodologies and classifications, the most relevant qualitative and quantitative results with their possible units and the most common interpretations
- Pertinence of each listed observation to other types of microorganism

In an iterative process, the initial list of data elements was modified to reflect the discussions among experts resulting in the exclusion of some IC items that were not strictly laboratory-related and in the incorporation of new items that had not been considered in the mentioned use case. For each listed observation we checked within the group if all the most commonly used qualitative, quantitative and semi-quantitative results were included. If this was not the case, we proceeded with the introduction of new items in the data set. In a similar pattern we moved forward also with units and interpretation of results. The analysis of the listed methodologies highlighted the need to expand the data set on microbial genetics.

In general, while the IC data set was tailored for a specific use case, the CDS module was expected to represent any possible relevant information concerning microbiology and thus required a more general modeling approach. For example, in HiGHmed, a specific list of bacteria, viruses and fungi was targeted, whereas the MII module was meant to address unlimitedly all organisms, including prions^73,74^, that could be of potential interest for a microbiology laboratory and of relevance for the field of infectious diseases. We therefore explored which microorganisms could conceivably be targeted by the listed different diagnostic methodologies. By doing so, soon it appeared clear that most tests are applicable to different categories of microorganisms (e.g., culture can be used for bacteria, viruses and fungi). We consequently decided to organize the information into three major blocks according to the main diagnostic methods used in the microbiology laboratories: culture (including microscopy), molecular diagnostics, and serological and immunological techniques. HELP and ELGA were mainly used as reference for the structure and the syntax of individual elements. HELP in fact focuses on specific bacterial infections and ELGA, while offering an important data model for laboratory findings, is based on the CDA standard^23^ and is designed for the Austrian healthcare.

During the working group meetings, also terminology bindings were examined and discussed until an internal agreement was reached. In parallel, the resulting module elements were entered into ART DECOR, a platform which is particularly useful for sharing data models as it supports the association of the data elements to standard terminologies. We were thus able to add the agreed codes and VSs to the platform.

Following the recommendations by the MII steering committee, we prioritized the international terminologies LOINC, SNOMED CT and UCUM to represent the elements of our data model. Working group members specialized in clinical terminology identified code systems and codes most suitable for application. In general, we followed the advice provided by the standard organizations on the combined use of SNOMED CT and LOINC for laboratory examinations. They suggest that the test performed should be coded with LOINC whereas the qualitative or nominal result be coded with SNOMED CT. Code selection was largely conducted via two web-based interfaces: the SNOMED CT Browser^75^ (version 2023.4.30) by SNOMED International and the SearchLOINC^76^ application (version 2.74) by Regenstrief Institute. If we found appropriate codes for a data element, we discussed the results within the group meetings and defined a corresponding VS, reflecting any changes identified as necessary by the microbiology experts. If no appropriate codes were found, further steps were discussed in the meetings as well. Under these circumstances, we evaluated three alternatives, such as choosing a different terminology system, submitting new code requests to the standard terminology organizations, or creating a new Code System, and applied each alternative on a case-by-case basis.

FHIR VSs and, if necessary, self-defined CodeSystems were created with the web-based Snapper^77^ application (version 2.7.0) according to the naming conventions defined within the MII. Thanks to the application’s integration with the terminology server Ontoserver^78^, an integrated validation and trial expansion of each defined VS was facilitated. Likewise, the terminology experts mutually validated their work. To create intensional VSs, SNOMED CT offers the possibility to use an Expression Constraint Language (ECL)^79^ to define bounded sets of concepts that satisfy a specific constraint^80^. For example, to create an intensional VS of all organism concepts in SNOMED CT we could use the ECL expression:

<410607006 |Organism (organism)|

To create intensional VS with LOINC, which, differently from SNOMED CT, is based on the combination of multi-axial LOINC parts (e.g., Property, Method, Class) instead of the description logic definitions, we applied rules using the parts. For example, to select the appropriate codes for phenotypic susceptibility, we have used the following rule: all LOINC codes that refer to phenotypic methods and that have Property equal “susc”, which stands for “susceptibility”. After the content and the organization of the information was established, we could proceed with the modeling of the information in the HL7 standard FHIR format version R4 using the Forge^81^ desktop application (version 30.2.0). With Forge, it is possible to have a visual interface to customize the FHIR resources to specific needs.

### FHIR profiling

The microbiology-related information, consisting of laboratory examinations, was built using the Observation and the DiagnosticReport resources. To represent all the information included in the microbiology module, several separate FHIR profiles were customized as different adaptations of the basic resource. The profiles developed to describe the observations all share the same principal structure with the data elements of particular relevance being summarized in Table 11 together with a description of their meaning in the module’s context.

**Table 11:** The most relevant FHIR elements of the Observation resource used to describe microbiology examinations.

FHIR Observation element	Use
Observation.code	Examination performed
Observation.value	Numerical, nominal or ordinal result
Observation.interpretation	Interpretation of the result obtained
Observation.component.code	Possible further specification of the same examination leading to an additional result
Observation.component.value	Additional results from the same examination
Observation.hasMember	Relation to another examination
Observation.method	Method used to perform the examination

The description of the examination performed was modelled in *Observation.code* whereas the corresponding result is defined in *Observtion.value*. As a general pattern, tests investigating the presence of microorganisms, antibodies, antigens or genetic material were all modelled with a qualitative result in *Observation.value*. Possible additional numerical results were coded as *Observation.component*. Examinations that further characterize the properties of a detected microorganism, such as the colony count or anti-microbial susceptibility testing, were linked together via the FHIR element *Observation.hasMember*.

While these principal elements appear repeatedly throughout the data model, they are each time adapted to the specific needs in the respective context. In particular, the previously selected terminology codes were associated to the corresponding profiles elements. Likewise, the FHIR globally unique identifier (canonical URL) were used to associate the created VSs.

To define the microbiology report type, the *DiagnosticReport.category* element of the base laboratory resource was customized.

In all profiling activities, the working group attempted to achieve the most efficient configuration to satisfy the different laboratories’ requirements while at the same time facilitating data exchange and integration.

Finally, both FHIR profiles and VSs were uploaded to the Simplifier platform to be publicly available to the scientific community in both JSON and XML formats together with an Implementation Guide.

### Supplementary information

Supplementary Figure 1

## Data Availability

The microbiology module data set is available on the standard-enabling platform ART-DECOR in German and English (https://art-decor.org/ad/#/mide-/datasets/dataset/2.16.840.1.113883.3.1937.777.24.1.1/2018-06-05T12:44:12/concept/2.16.840.1.113883.3.1937.777.24.2.73/2018-06-07T16:58:36) and in the Figshare repository 10.6084/m9.figshare.22799042^82^.

## Code availability

The profiles are available in version 1.0 on the FHIR collaboration and publishing platform Simplifier: https://simplifier.net/medizininformatik-initiative-modul-mikrobiologie and in the Figshare repository 10.6084/m9.figshare.22799042^82^.
