# Author Correction: Towards interoperability in infection control: a standard data model for microbiology

**DOI:** 10.1038/s41597-023-02853-1

**Published:** 2023-12-20

**Authors:** Eugenia Rinaldi, Cora Drenkhahn, Benjamin Gebel, Kutaiba Saleh, Hauke Tönnies, Friederike D. von Loewenich, Norbert Thoma, Claas Baier, Martin Boeker, Ludwig Christian Hinske, Luis Alberto Peña Diaz, Michael Behnke, Josef Ingenerf, Sylvia Thun

**Affiliations:** 1 Berlin Institute of Health, Charité Universitätsmedizin, Berlin, Germany; 2 Institute of Medical Informatics (IMI), University of Lübeck, Lübeck, Germany; 3 Klinik für Infektiologie und Mikrobiologie, Universitätsklinikum Schleswig-Holstein, Campus Lübeck, Lübeck, Germany; 4 Data Integration Center, Jena University Hospital, Jena, Germany; 5 Universitätsklinikum Münster, Münster, Germany; 6 Institute of Virology, University of Mainz, Mainz, Germany; 7 Institute for Hygiene and Environmental Medicine, Charité Universitätsmedizin, Berlin, Germany; 8 Hannover Medical School, Institute for Medical Microbiology and Hospital Epidemiology, Hannover, Germany; 9 Technische Universität München, München, Germany; 10 Institute for Digital Medicine, University Hospital Augsburg, Augsburg, Germany

**Keywords:** Medical research, Health care

Correction to: *Scientific Data* 10.1038/s41597-023-02560-x, published online 23 September 2023

In this article, the grant number relating to the German Federal Ministry of Education and Research was not properly acknowledged. The original article has been corrected.

