# Supplementary material for: Towards interoperability in infection control: a standard data model for microbiology
Source: Sci Data. 2023 Sep 23;10:654. doi: 10.1038/s41597-023-02560-x (PMC10517923; doi:10.1038/s41597-023-02560-x)
Supplement: Supplementary file 1 — Supplementary Figure 1 [file 41597_2023_2560_MOESM1_ESM.pdf]

Table of contents:

Supplementary Figure 1

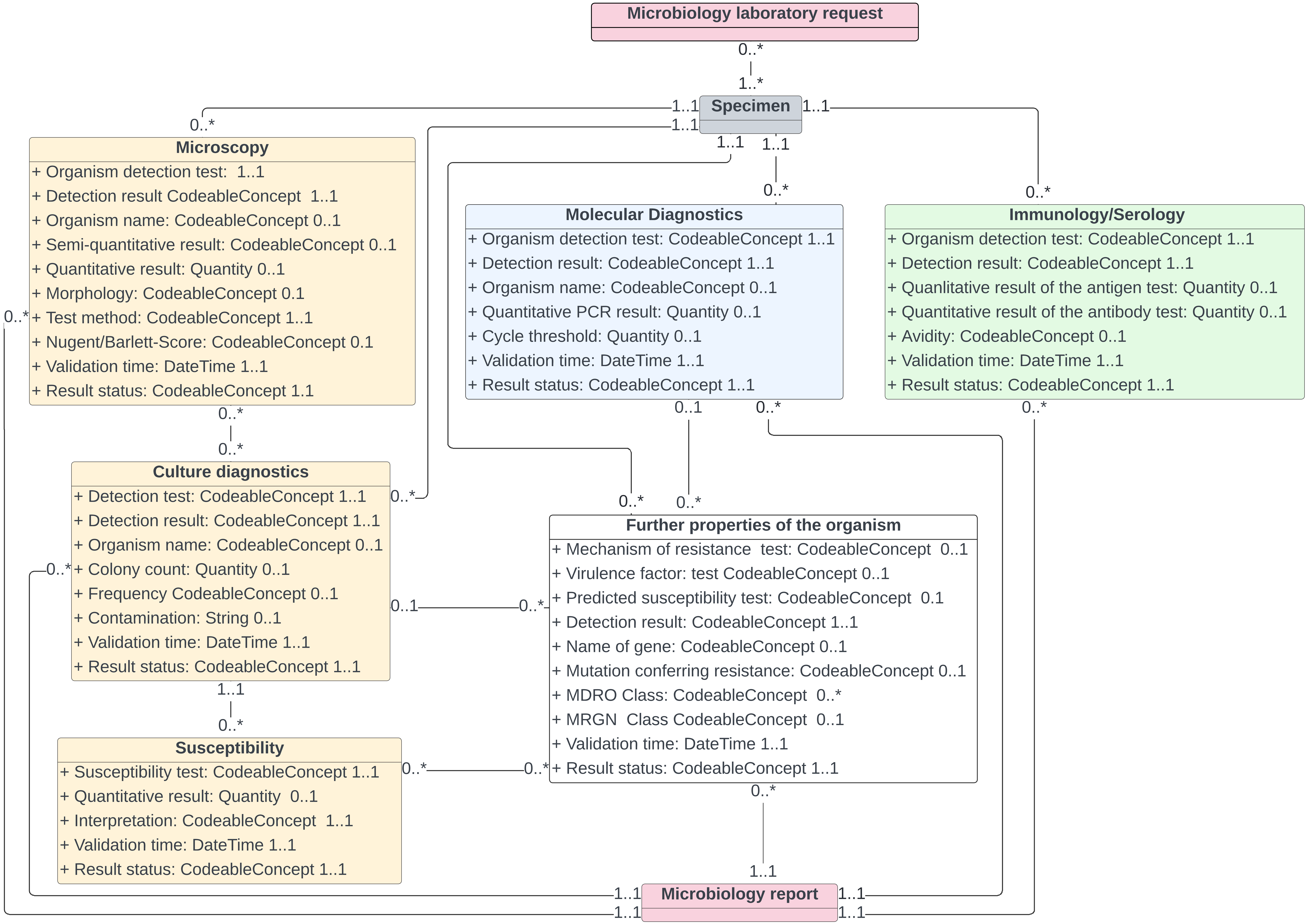


Supplementary Figure 1: UML Diagram for microbiology information.
